# Mono- and multilayers of molecular spoked carbazole wheels on graphite

**DOI:** 10.3762/bjoc.10.295

**Published:** 2014-11-27

**Authors:** Stefan-S Jester, A Vikas Aggarwal, Daniel Kalle, Sigurd Höger

**Affiliations:** 1 Kekulé-Institut für Organische Chemie und Biochemie, Rheinische Friedrich-Wilhelms-Universität Bonn, Gerhard-Domagk-Str. 1, 53121 Bonn, Germany

**Keywords:** molecular spoked wheels, scanning tunneling microscopy, self-assembled monolayers, solid/liquid interface, template

## Abstract

Self-assembled monolayers of a molecular spoked wheel (a shape-persistent macrocycle with an intraannular spoke/hub system) and its synthetic precursor are investigated by scanning tunneling microscopy (STM) at the liquid/solid interface of 1-octanoic acid and highly oriented pyrolytic graphite. The submolecularly resolved STM images reveal that the molecules indeed behave as more or less rigid objects of certain sizes and shapes – depending on their chemical structures. In addition, the images provide insight into the multilayer growth of the molecular spoked wheels (MSWs), where the first adlayer acts as a template for the commensurate adsorption of molecules in the second layer.

## Introduction

**Figure 1:**
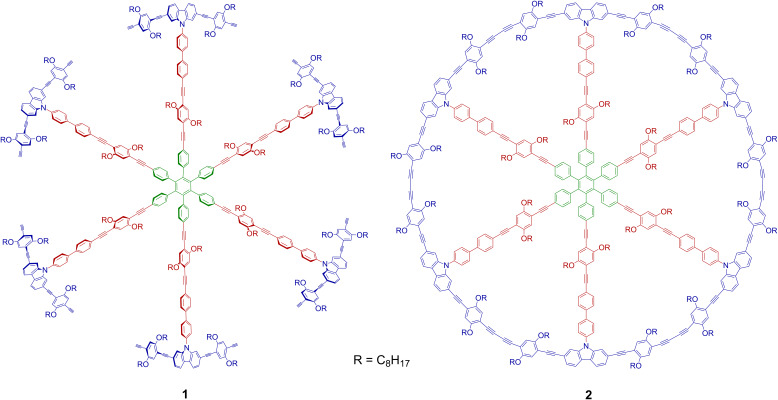
Chemical structures of precursor **1** and MSW **2**. Hub, spokes, and rim units are shown in green, red, and blue colors, respectively.

Molecular spoked wheels (MSWs) have gained increasing interest as two-dimensional (2D) carbon-based objects of adjustable sizes [1–5]. They can be viewed as shape-persistent arylene–alkynylene macrocycles in which the intraannular spoke system increases the stiffness (persistence length) of the ring. They are non-collapsible monodisperse cyclooligomers with a fixed and predictable conformation, and their side-chain substitution guarantees their solubility in organic media. Our previous works on freely rotating chains of rigid rod segments and on shape-persistent macrocycles [6–7] have recently led us to a set of molecular polygons (macrocycles) of discrete sizes and symmetries, e.g., triangles, squares, pentagons, and hexagons [8]. These represent basic building blocks for a supramolecular Archimedean surface tessellation system [9]. Thereby, the highly oriented pyrolytic graphite (HOPG) acts as a template along the main axis directions of which the alkyl/alkoxy side chains align [10–11], and consequently the superstructures can be viewed as commensurably aligned adlayers. Among a series of characteristic superstructures, a hexagonal pattern is observed for molecular hexagons that self-assemble at the liquid/solid interface of 1,2,4-trichlorobenzene (TCB) and HOPG, however, the molecules tend to collapse (by rotation around the single bonds of two corner units) in the presence of molecular squares [8]. Consequently, an increased understanding of the 2D self-assembly of MSWs on HOPG should nominally pave the way towards a suitable molecular design for (stiffened) hexagons that is compatible with our previous series and might form cocrystals with other polygons, thus patterns of increased complexity and larger lattice constants become feasible. Our MSW **2** and its precursor **1** were recently investigated by means of single-molecule photoluminescence spectroscopy as model compounds for conjugated oligomers commonly used in polymer light emitting diodes [12]. The chemical structures of both compounds are shown in Figure 1. Their synthesis and characterization has been reported before [11]. Here, we present scanning tunnelling microscopy (STM) investigations of both compounds, aiming at an extended description of the observed molecular geometries and their supramolecular monolayer and multilayer formation on HOPG.

## Results and Discussion

We started our STM investigations on the MSW **2** using TCB as a standard solvent for STM, but did not observe molecules, most probably because they do not adsorb to form two-dimensional (2D) supramolecular adlayers at the liquid/solid interface of TCB and HOPG. When using 1-phenyloctane, another commonly used solvent for STM measurements, we could acquire some images of the MSWs, but imaging was less stable (Supporting Information File 1). However, much better images of **2** were obtained using a third solvent, octanoic acid (OA), which has also previously been used for imaging MSWs [2,4]. We compared the so-obtained images of MSW **2** with its precursor **1** under similar conditions. In all images, regions covered with conjugated backbones and alkoxy side chains are observed in bright and dark colors, related to high and low tunneling currents, respectively [13].

**Figure 2:**
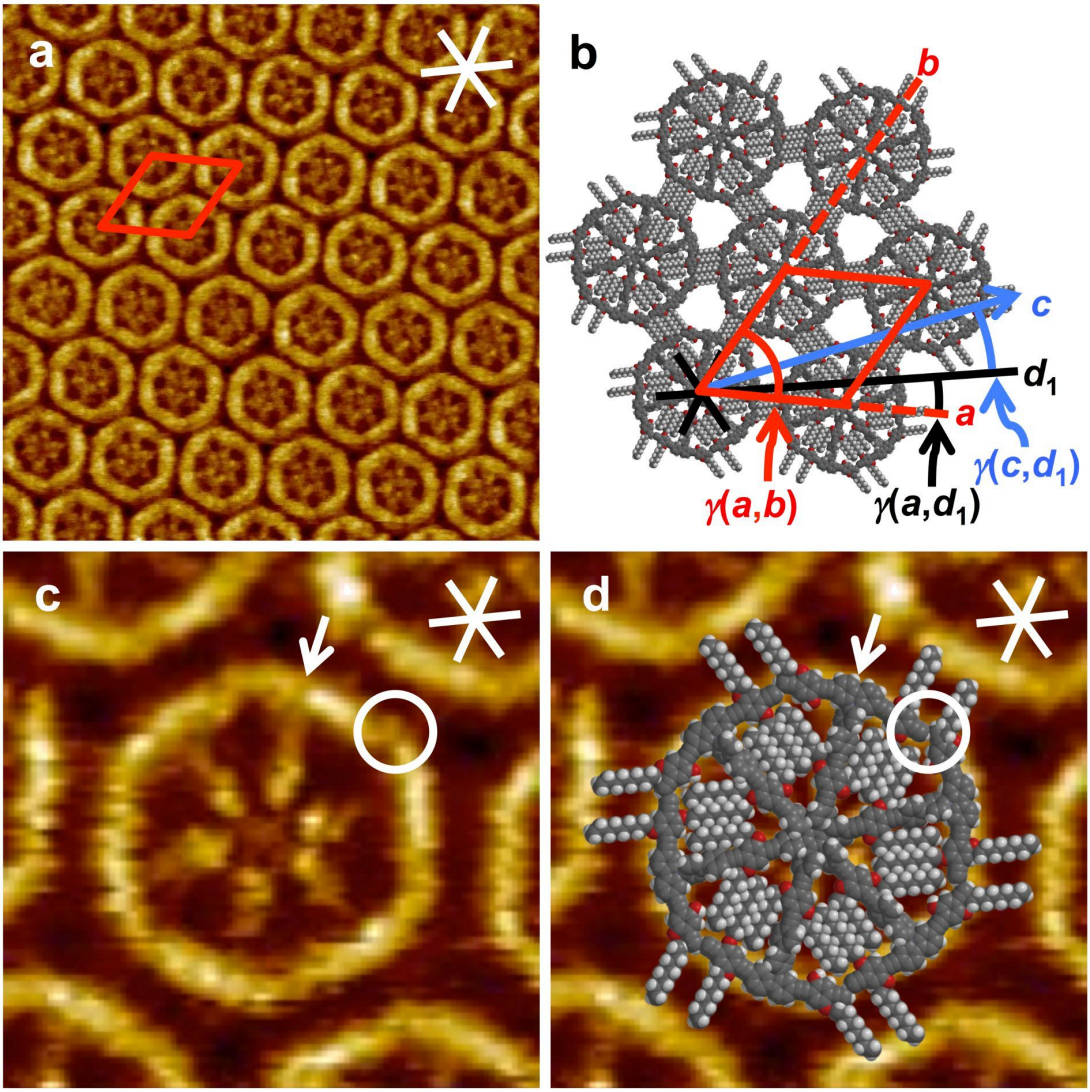
(a)–(d) STM images, a molecular, and a supramolecular model of **1** at the OA/HOPG interface. (a) STM image (40.9 × 40.9 nm^2^, *V*_S_ = −1.6 V, *I*_t_ = 5 pA) and (b) supramolecular model: The MSW precursors self-assemble into a 2D-crystalline pattern of densely packed molecules. A hexagonal unit cell (red lines) of *a* = *b* = 6.7 ± 0.2 nm, γ(*a*,*b*) = 60 ± 2° is indexed. The unit cell vector *a* is aligned relative to one of the three HOPG main axis directions (black, white lines), *d*_1_, with γ(*a*,*d*_1_) = 9 ± 2°. The direction of the backbones, *c*, as defined by two spokes (and shown by the blue arrow), is *γ*(*c*,*d*_1_) = 12 ± 2°. (c) and (d): High-resolution STM image (9.3 × 9.3 nm^2^, *V*_S_ = −1.6 V, *I*_t_ = 4 pA), (c) without and (d) with a superimposed molecular model. The white arrows indicate one representative carbazole unit that connects a rim segment with a spoke. The white circles indicate one representative kink of the brightly appearing hexagon, and the corresponding region in the molecular model where two adjacent rim segments meet. In other words, the kinks do not correspond to the carbazole units, but originate from adjacent rim segments that align in a characteristic fashion. Both STM images were acquired from a 2 × 10^−6^ M solution of **1** in OA, and the sample was thermally annealed for 30 s at 70 °C prior to imaging.

At the OA/HOPG interface **1** forms a 2D-crystalline monolayer (Figure 2a and b) for which a hexagonal unit cell of *a* = *b* = 6.7 ± 0.2 nm, γ(*a*,*b*) = 60 ± 2° can be indexed. The unit cell vector *a* is oriented with γ(*a*,*d*_1_) = 9 ± 2° relative to one of the HOPG main axes (and alkoxy side chain alignment directions) [10–11], *d*_1_*.* A high resolution STM image is shown in Figure 2c, and a molecular model is superimposed to a copy of the image in Figure 2d. On a first sight, a bright hexagonal frameline (corresponding to the six rim segments) is observed, which is filled with a star-shaped (spoke) system and central darker spot (hub; cf. definition in Figure 1). In other words, the rim units appear (mostly) as a continuous line. The six rim segments contain central carbazole units (cf. chemical structure in Figure 1), so that they are only slightly curved (or bent; as seen in the molecular model in Figure 2c and d where one of the six carbazole units is marked by a white arrow). They are still not directly connected, but terminated with acetylene units. However, a gap can rarely be estimated from the brightness modulation (cf. circle in Figure 2c). Moreover, the six kinks that are seen in the STM images (one of which is marked by the white circle in Figure 2c and d) correspond (not to the carbazole units but) to the intersections of the terminal acetylenes of the rim segments.

According to the space-filling model, only three of the four pseudo-intraannular alkoxy side chains fit (after adsorption) into the triangular cavity regions between each two spokes and the rim. Based on the observed intermolecular distances and an alignment of the alkoxy side chains along the HOPG main axis directions [10–11], an intermolecular side chain interdigitation scheme as shown in Figure 2b is proposed. Alternatively, it cannot be excluded that all intraannular alkoxy chains point to the solution phase. Due to the high flexibility and the weak electrical conductivity of the alkoxy chains, this commonly does (rather) not affect the image quality if the molecules are sufficiently strong bound to the substrate. Oppositely, the pseudo-extraannular side-groups are most probably adsorbed on the graphite surface and interdigitate, since the observed (regular) distance between the molecules fits perfect with the molecular model. Additionally, this assumption is supported from the slight contrast undulation between the backbones in the STM image.

**Figure 3:**
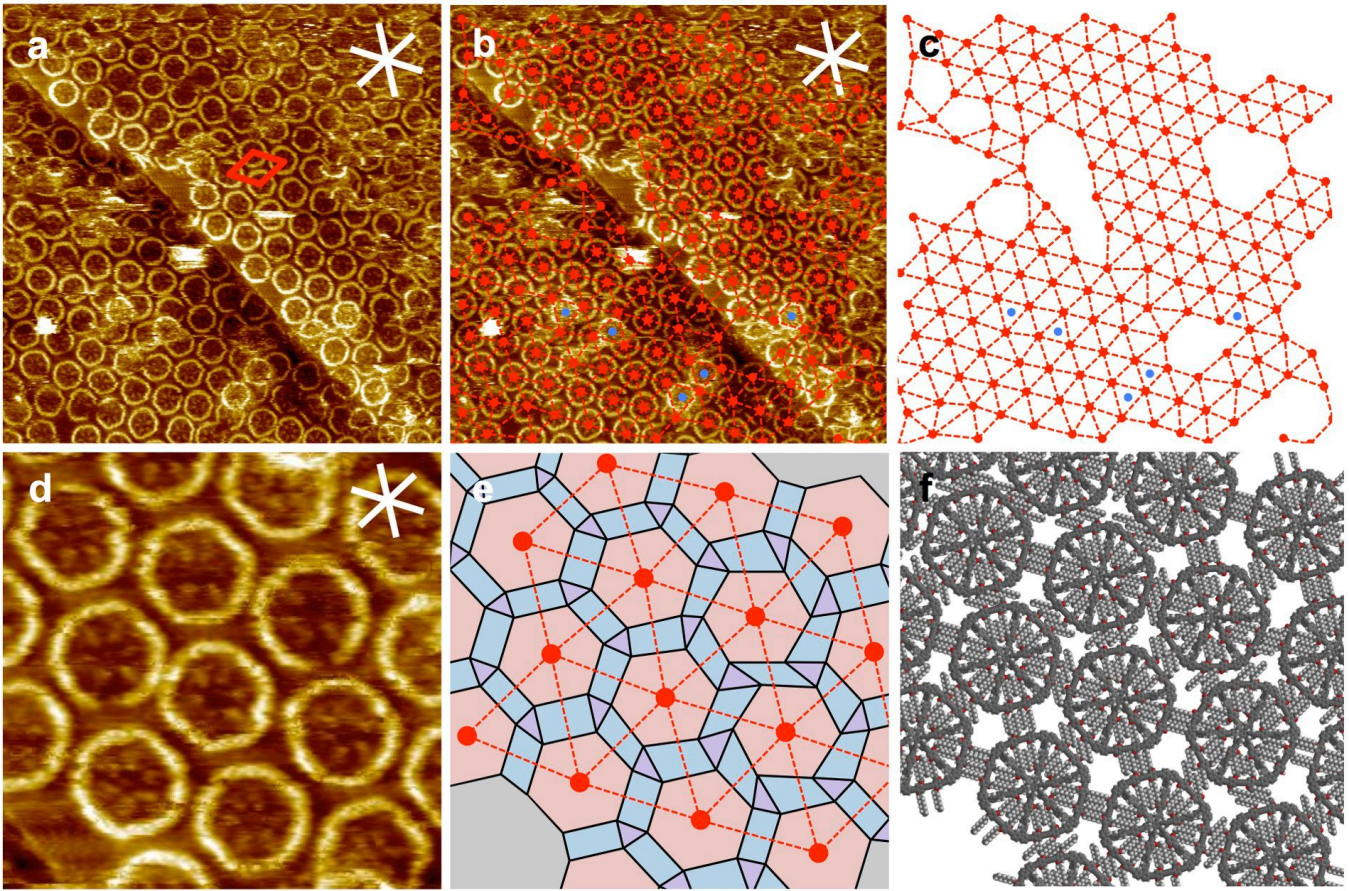
(a)–(f) STM images, mesh, polygon, and supramolecular models of MSW **2** at the OA/HOPG interface. (a) The overview STM image (88.6 × 88.6 nm^2^, *V*_S_ = −1.5 V, *I*_t_ = 10 pA) shows an adlayer in which the circular shapes of the MSW rims and features of the central hub/spoke system are recognizable. A (nominal) unit cell of *a* = *b* = 6.9 ± 0.2 nm, γ(*a*,*b*) = 60 ± 2° (red lines) is approximated. (b) A copy of the STM image shown in (a), where the centers of all MSWs are marked by red dots, and dashed lines are drawn to connect all adjacent dots, so that a network structure results, which is shown without the STM image in (c). (d) A more detailed STM image (23.0 × 23.0 nm^2^, *V*_S_ = −1.5 V, *I*_t_ = 10 pA) confirms the high variation of rim–rim distances. (e) A mesh model is drawn similar as in (b). The centers of equilateral hexagons are placed at the positions of the red dots, and the orientation of each hexagon is fitted to match the MSW orientation (by rotation). The corners of adjacent hexagons are connected by solid black lines to form triangles and tetragons, the variation of the shapes of which points out the high degree of disorder. (f) The data obtained in (d) and (e) is translated into a supramolecular model. The alkoxy side-chains are subsequently added to match the expected directions along the HOPG main axes [10–11], showing that the intermolecular interactions cannot be described by (only) one side-chain interdigitation motif. The MSWs were deposited from a 10^−5^ M solution, and the sample was thermally annealed for 30 s at 80 °C.

Opposed to **1**, MSW **2** self-assembles into a less ordered adlayer (Figure 3a). Throughout all STM images, the expected MSW-like shape of each of the molecular entities is clearly apparent. The conjugated rims appear brightly, but have a significantly more round and/or disturbed shape (Figure 3d) as compared to the precursor **1** (Figure 2c). The spoke units of the MSW (Figure 3a and d) appear – throughout all images – less brightly than the rim, which was not the case for precursor **1** (Figure 2a, c, d). The central hub does nearly show any (bright) tunneling current features. For the overall hexagonal packing a (nominal) unit cell of *a* = *b* = 6.9 ± 0.2 nm, γ(*a*,*b*) = 60 ± 2° is approximated where both unit cell vectors are aligned along the HOPG main axis directions (Figure 3a). The centers of all MSWs in the STM image in Figure 3b (which is a copy of the image shown in Figure 3a) are marked by red dots. All adjacent dots are connected by dashed lines, resulting in a mesh model of triangular tiles and sixfold vertices (Figure 3c). However, following the dashed lines along each of the six predominant directions shows a slight zig-zagged distortion. The mesh has a significantly lower degree of order than expected for a 2D-crystalline packing (that was observed for **1**). Some regions even do not allow a detailed interpretation (as shown as blank parts in Figure 3c).

A higher resolved STM image (shown in Figure 3d) shows that the MSW distances are not equal. The MSW centers are again marked by red dots and connected by dashed red lines (to form the red mesh shown in Figure 3e). The center of an equilateral hexagon is placed onto each of the red dots in Figure 3e, and the orientation of each hexagon is fitted (by rotation) to match the orientation of each corresponding MSW observed in Figure 3d. In other words, the high-resolution STM image is transcribed into a polygon model. The corners of adjacent hexagons are connected with solid black lines. The resulting triangles and tetragons are far from having identical shapes that were expected for a crystalline pattern (for two hypothetical packings and corresponding tessellation patterns, see Supporting Information File 1). The STM image (shown in Figure 3d) and the mesh model (shown in Figure 3e) are transcribed into a supramolecular model (shown in Figure 3f) based on rigid (ideal) backbones and subsequently added alkoxy side chains, each aligned along the HOPG main axis direction it fits best to (cf. white lines in Figure 3d) [10–11]. The result confirms that the intermolecular interaction cannot be described by a single packing concept. Consequently, the packing must be interpreted as an effect of a variety of different intermolecular (and intramolecular) alkoxy side-chain interaction/interdigitation motifs. The degrees of freedom of each MSW **2** are reduced (as compared to **1**), and the lower flexibility of the MSWs together with their large size and the resulting molecule–surface interaction decrease the ability to form a regular pattern, probably (also) due to a lower compound solubility. We observed a similar behavior for phenylene–ethynylene–butadiynylene macrocycles which we compared to their acyclic analoga of identical oligomerization degree [7].

**Figure 4:**
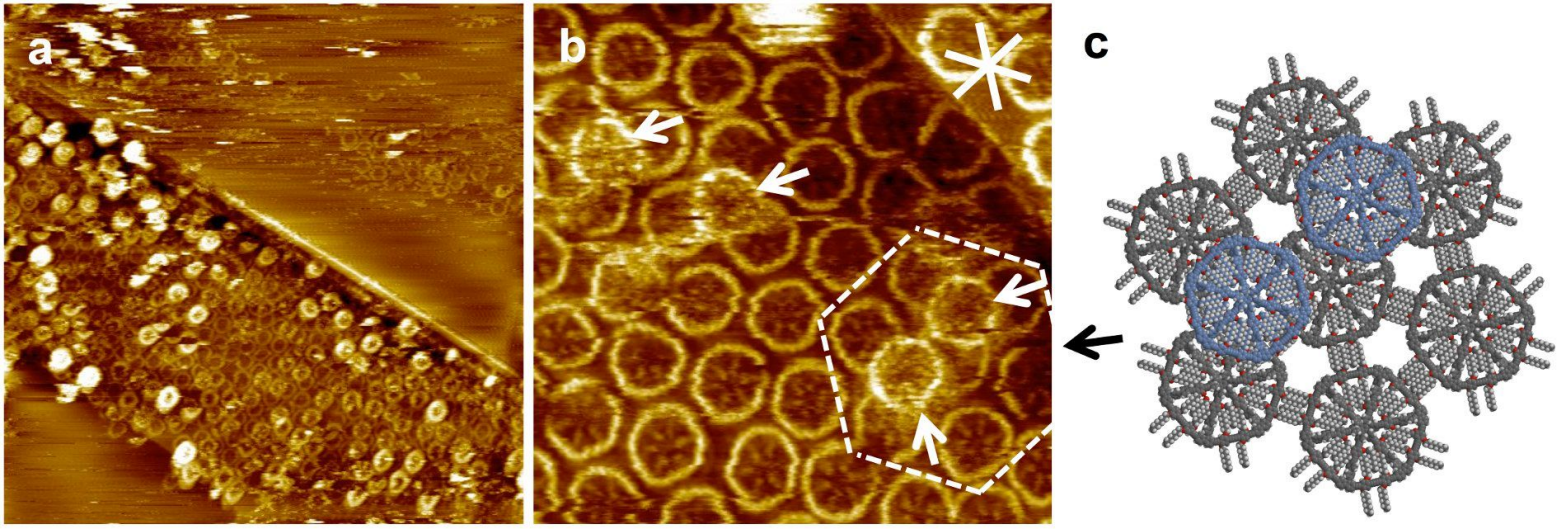
(a)–(c) STM images and a supramolecular model of MSW **2** at the OA/HOPG interface. (a) Overview STM image (200 × 200 nm^2^ (internal scanner calibration), *V*_S_ = −1.4 V, *I*_t_ = 5 pA). A several 10 × 10 nm^2^ large region is covered by a monolayer and (partly) a multilayer, whereas large parts remain uncovered. (b) The more detailed STM image (40.5 × 40.5 nm^2^, *V*_S_ = −1.5 V, *I*_t_ = 10 pA) shows MSWs that are adsorbed in a second layer on top of the centers of three supporting molecules (and indicated by white arrows). (c) Supramolecular model of the region marked by white dashed lines in (b). The first MSW monolayer (on HOPG) is shown in grey color, whereas the molecules in the second layer are shown in blue color (and their pseudo-extraannular alkoxy side chains are not shown). The molecules were adsorbed from a 10^−5^ M solution, and the sample was thermally annealed for 30 s at 80 °C prior to imaging. The white asterisk in (b) indicates the HOPG main axis directions.

Moreover, a strong tendency of **2** to stack into multilayers was observed, even if the first adlayer was not fully covered with MSWs (as seen in the overview STM image in Figure 4a). The more detailed STM image in Figure 4b shows that the MSWs in the second layer adsorb on top of the center between three first-layer MSWs (cf. arrows in Figure 4b, and the underlying molecular model in Figure 4c that represents the region marked by the dashed lines in Figure 4b). The central hub of the second-layer MSWs, which can for sterical reasons not planarize, fits perfectly into the intermolecular cavities formed by three adjacent molecules in the first layer. In addition, the MSWs in the second layer in the region marked by the white dashed lines in Figure 4b are rotated by ~30° relative to the MSWs in the first layer, as seen from the orientation of the rim segments, so that their pseudo-extraannular octyloxy side chains are (most probably) adsorbed on the HOPG substrate in the intermolecular pores of the first layer. The results clearly indicate that not only the HOPG acts as a template for the organization of the first molecular adlayer, but the adsorption pattern is transferred to the second layer. In other words, the first layer of MSWs in a template for the commensurate adsorption of the second MSW layer.

In STM experiments, the second adsorption layer is generally not observed due to the high mobility of the molecules therein with respect to the scanning STM tip, and the higher tunneling resistivity. Coadsorption on a (supra-)molecular template is rather an exception than a rule. However, when observed, it is often between electron-rich macrocycles that act as hosts for electron-deficient guest molecules (e.g., fullerenes, metallacycles) [14–15]. In the case here, the adlayer stability can be ascribed to a mechanical interlocking together with a high van der Waals interaction due to the large molecule size. It is worth to note that these investigations may also give insight into the spoked wheel organization in the bulk material.

## Conclusion

Precursor **1** and MSW **2** self-assemble at the OA/HOPG interface into 2D adlayers. In both cases, the packing is determined by the molecular backbone shapes and the attached octyloxy side chains that tend to pack densely and align along the main axis directions of the HOPG substrate which acts as a template. The STM images of both, **1** and **2**, reveal the differences in the molecular structures that result in different adsorption behavior. While only 2D-crystalline monolayers were observed for the more flexible precursor **1**, MSW **2** forms less ordered patterns and tends to form multilayers. A characteristic stacking of the macrocycles in the second layer on top of three macrocycles of the first layer is observed, showing that the first molecule layer acts as a template for the second molecule layer.

## Experimental

The synthesis and characterization of the compounds has been reported before [11]. STM was performed at the liquid/solid interface under ambient conditions. Typically, 0.5 µL of a 10^−5^–10^−6^ M solution of the respective substance in OA was dropped onto a piece of freshly cleaved HOPG at elevated temperature (70–80 °C), and the sample was allowed to cool to rt prior to STM imaging. All STM measurements were performed in situ (with the tip immersed into the liquid) and typically completed within 30 min after the sample preparation. Bias voltages between −1.4 V and −1.6 V and current setpoints between 4 pA and 10 pA were applied to image the molecular adlayers shown in this work. Mechanically cut Pt/Ir (80:20) tips were used and further modified (while imaging) by applying short voltage pulses. All STM images were calibrated by subsequent immediate acquisition of an additional image at reduced bias voltage, therefore the atomic lattice of the HOPG surface is visible, which is used as a calibration grid. Data processing, also for image calibration, was performed using the SPIP 5 (Image Metrology) software package.

## Supporting Information

File 1 Theoretical considerations on supramolecular pattern structures of **2** and additional STM images.
