# Supplementary material for: Mono- and multilayers of molecular spoked carbazole wheels on graphite
Source: Beilstein J Org Chem. 2014 Nov 27;10:2783–8. doi: 10.3762/bjoc.10.295 (PMC4273219; doi:10.3762/bjoc.10.295)
Supplement: File 1 — Theoretical considerations on supramolecular pattern structures of 2 and additional STM images. [file Beilstein_J_Org_Chem-10-2783-s001.pdf]

Supporting Information

for

Mono- and multilayers of molecular spoked carbazole wheels on graphite

Stefan-S. Jester*, A. Vikas Aggarwal, Daniel Kalle and Sigurd Höger

Address: Kekulé-Institut für Organische Chemie und Biochemie, Rheinische Friedrich-Wilhelms-Universität Bonn, Gerhard-Domagk-Str. 1, 53121 Bonn, Germany

Email: Stefan-S. Jester - stefan.jester@uni-bonn.de

*Corresponding author

Theoretical considerations on supramolecular pattern structures of 2 and additional STM images

Conceptual approach to the packing of molecular spoked wheels

Molecular models of the molecular spoked wheel (MSW) **2** are shown in Figure S1a and e. The backbone scaffold is subdivided into a set of subunits as indicated in Figure S1a and b: Six *p*-phenylene–ethynylene–butadiynylene rim segments (rigid rods), six carbazole units, six *p*-phenylene–ethynylene spokes, and a central hexaphenyl benzene hub. The hexagonal shape of the rim is mechanically stabilized by the star-shaped spoke/hub system. The internal structure is subdivided into triangular compartments, each formed by a rim segment and two spokes as shown in Figure S1b.

Octyloxy substituents are added to the backbones in such a way that they are oriented in the plane defined by the backbone and adopt 60°/120°/180° relative angles (as considering a typical alignment of the adsorbed alkyl/alkoxy side chains along the main axis directions of the highly oriented pyrolytic graphite (HOPG) substrate) [S1]. Considering the length of the side chains and the pore sizes, at most three of the four side chains per triangular cavity can fill the pore, whereas at least the fourth side chain must point towards the third dimension (into the solution phase) and is not shown in the models here. The alkyl/alkoxy side chains are flexible and tend adopt an all-*trans* configuration. Two models including two different side chain alignments are shown in Figure S1a–d and e–h, respectively.

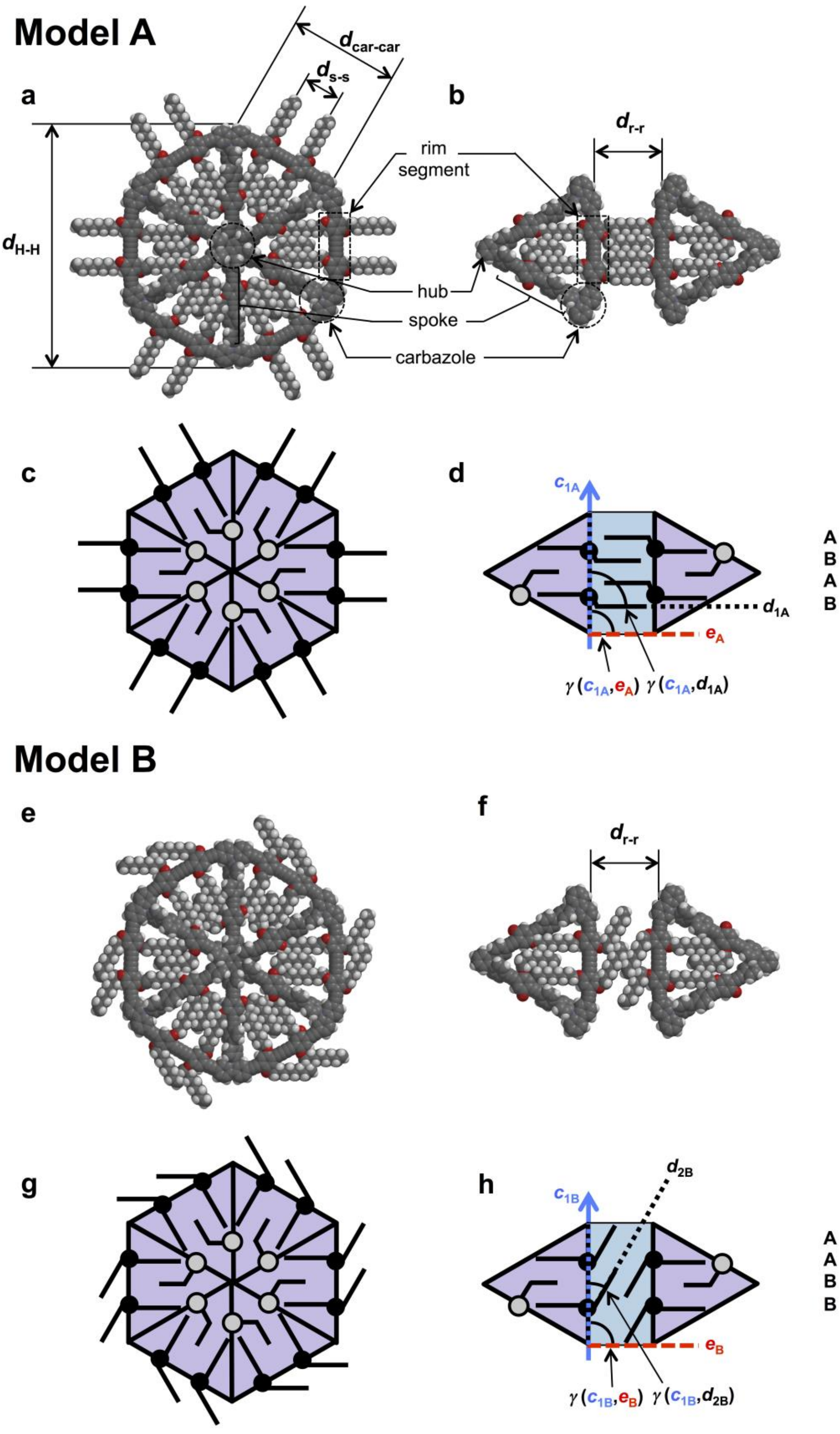

Figure S1. Molecular and schematic models of MSW **2** including different alkoxy side chain interdigitation schemes. (a) Molecular geometry of the backbone and octyloxy side chains with 60°/120°/180° relative orientation, and two alkoxy-backbone angles for pseudo-extraannular side chains of (a) $\gamma(c_{1A},d_{1A})$ = 90°. Indicated lengths are the

MSW diameter, $d_{H\text{-}H}$, of 5.9 nm, the carbazole–carbazole distance, $d_{car\text{-}car}$, of 2.8 nm, the phenylene–phenylene distance, $d_{ph\text{-}ph}$, of 0.98 nm. A rim segment, a carbazole unit, a spoke, and the central hub are assigned. (b) At most, three of the four side chains per triangular cavity may align inside the pore, whereas the fourth side chain per triangular cavity must point towards the solution phase (and is not shown here). Two triangular parts of two adjunct MSWs visualize an intermolecular side chain interdigitation scheme. All side chains are oriented perpendicular direction to the rigid rods. (c) Schematic model of (a). (d) Schematic model of (b), indicating (here): $\gamma(c_{1A},e_A) = \gamma(c_{1A},d_{1A}) = 90°$. e_A is defined as a direction related to the relative orientation of adjacent rim segments. (e) A second model of the MSW is shown where the pseudo-extraannular side chains are aligned along a different direction, $\gamma(c_{1B},d_{2B}) = 30°$. (f) A second intermolecular interaction scheme is shown for two triangular segments. (g) Schematic model for (e). (h) Schematic model for (f), $\gamma(c_{1B},d_{2B}) = 30°$, $\gamma(c_{1B},e_B) = 90°$. In all schematic models, black dots symbolize dialkoxy substituted aromatic units where both side chains are aligned on the HOPG substrate, whereas the grey dots symbolize dialkoxy substituted aromatic units where one side chain is adsorbed and the other points towards the solution phase. Alkoxy side chains that do not contribute to the packing but are either randomly adsorbed or point into the solution phase are not shown.

Two hypothetical packing schemes are shown in Figure S2a–c and d–f, as related to the side chain interaction schemes in Figure S1a–d and e–h, respectively. Both examples in Figure S2 are consistent with a rim–rim distance of $d_{r\text{-}r} = 1.7$ nm between two rim segments. The first model (Figure S2a–c) shows an interaction of two triangular subunits via octyloxy side chains following an ABAB interdigitation scheme and $\gamma(c_1,d_1) = 90°$. The second model (Figure S2d–f) shows two triangular subunits interacting via side chains following an AABB alignment scheme with $\gamma(c_1,d_2) = 30°$.

To both models, unit cells of $a_n = b_n = 6.9$ nm, $\gamma(a_n,b_n) = 60°$, n = A,B can be indexed. In addition, both patterns can be described as a set of equilateral hexagons,

triangles, and rectangles, to form a (3.4.6.4) pattern according the nomenclature previously described [S2], that is adapted from the nomenclature used in discrete geometry [S3].

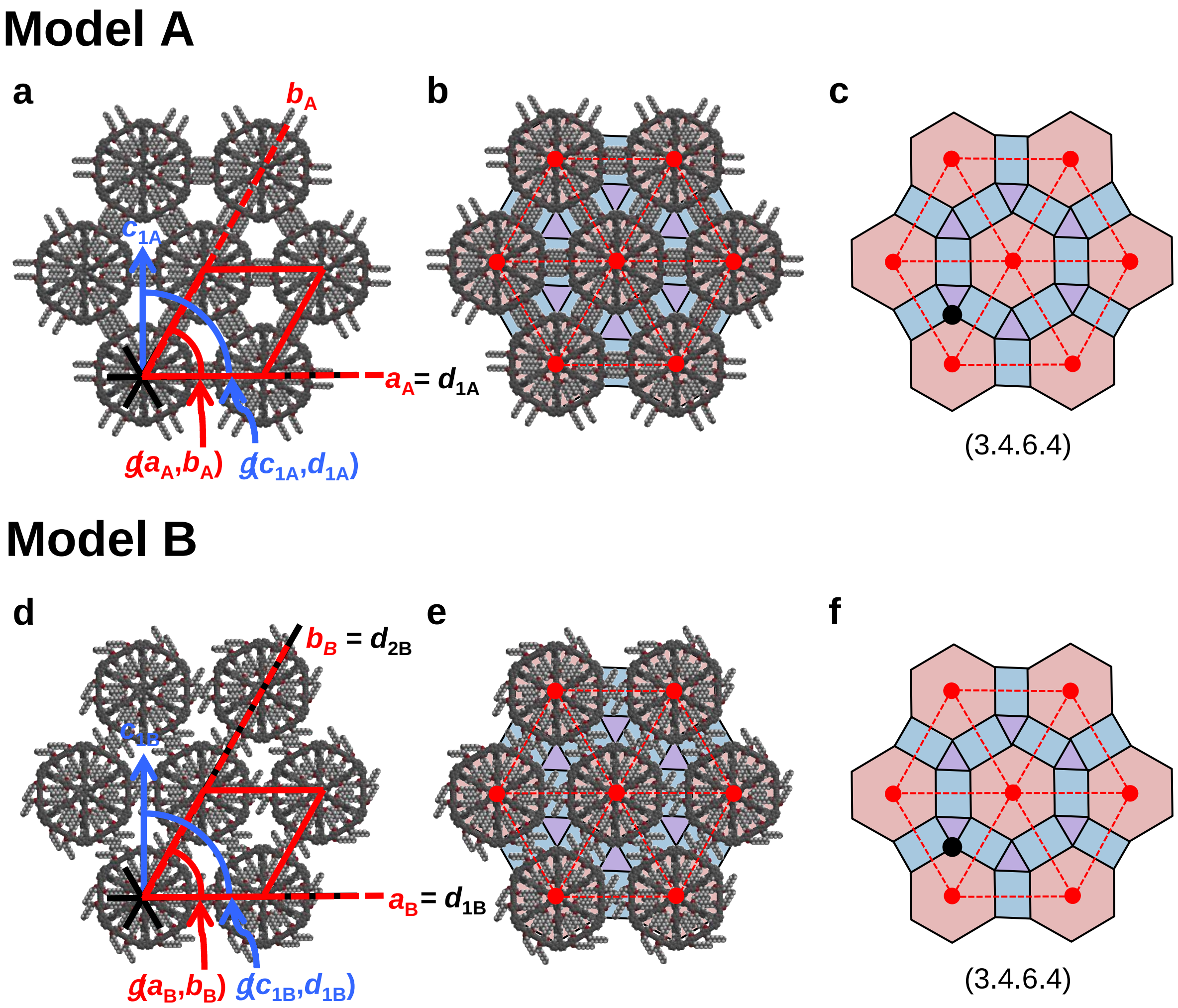

Figure S2. Two hypothetical packing schemes of **2** at the 1-octanoic acid/HOPG interface are shown which are based on the molecular models discussed in Figure S1. The predicted unit cells are $a_n = b_n = 6.9$ nm, $\gamma(a_n,b_n) = 60°$, n = A,B. (3.4.6.4) tilings can be assigned, in which the vertex is formed by a triangle, rectangle, hexagon, rectangle, in circular order [S2,S3].

Additional STM images

An STM image of an island rim of MSW **2** at the OA/HOPG interface is shown in Figure S3. In addition, the right part of the image a striped monolayer pattern of OA is observed. At the MSW island rim a reduced kinetical trapping of unfavorable packings is expected and the molecules should be able to rotate more freely, which should lead to a higher packing order. However when looking at the details, the MSW rims do not appear as perfect hexagons but slightly more round, and the relative orientation of the molecules slightly vary and lead to a reduced packing order.

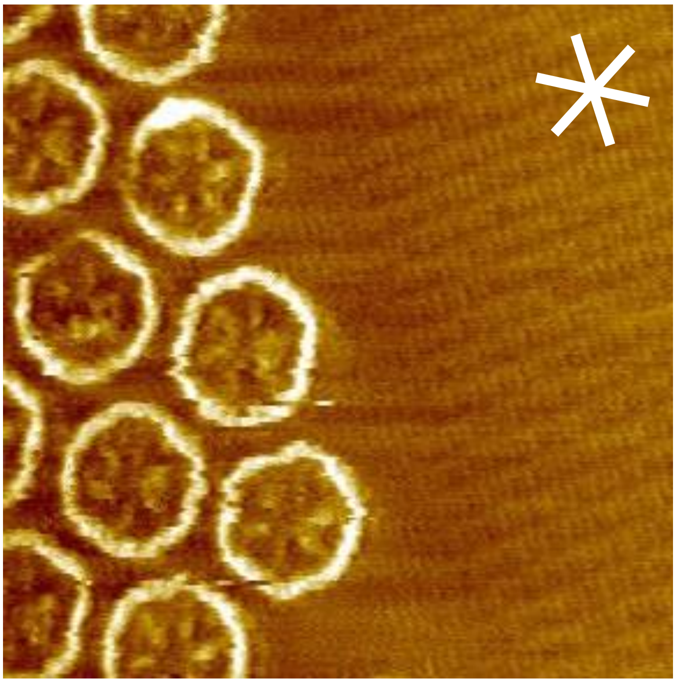

Figure S3. STM image of an island rim of MSW **2** at the OA/HOPG interface (26.7 × 26.7 nm^2, V_S = –1.5 V, I_t = 5 pA, c = 10^{-5} M, 20 s thermally annealed to 80 °C prior to imaging).

An additional STM image of MSW **2** at the 1-phenyloctane/HOPG interface is shown in Figure S4.

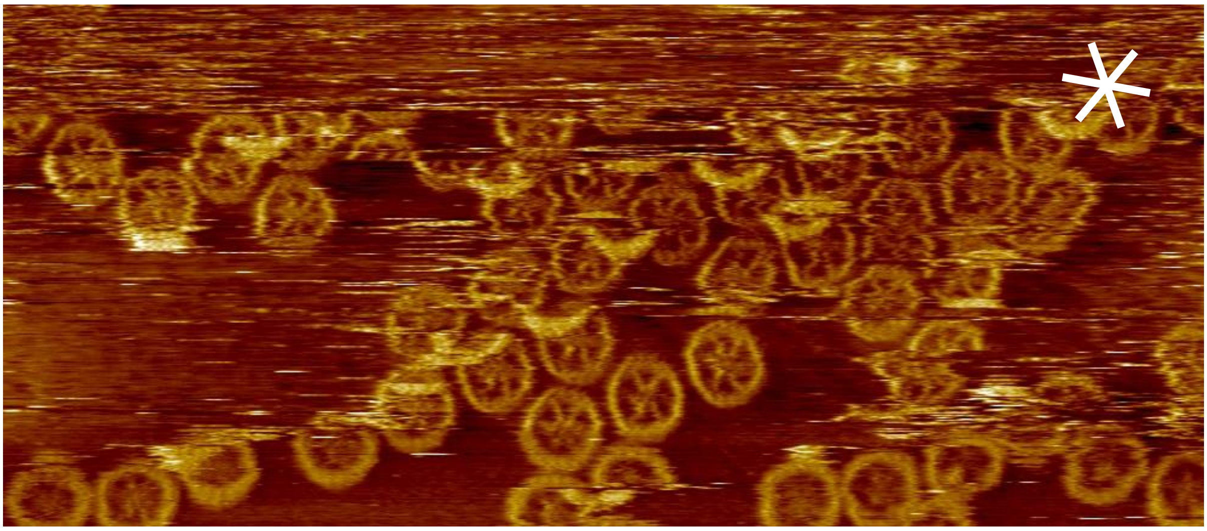

Figure S4. STM image of MSW **2** at the 1-phenyloctane/HOPG interface (38×89 nm^2, $V_S = -1.8$ V, $I_t = 2$ pA, $c = 2 \times 10^{-6}$ M, 30 s thermally annealed to 100 °C prior to imaging)

References

S1. a) Yang, T.; Berber, S.; Liu, J.-F.; Miller, G. P.; Tománek, D. *J. Chem. Phys.* **2008**, *128*, 124709; b) Ilan, B.; Florio, G. M.; Hybertsen, M. S.; Berne, B. J.; Flynn, G. W. *Nano Lett.* **2008**, *8*, 3160.

S2. Jester, S.-S.; Sigmund, E.; Röck, L. M.; Höger, S. *Angew. Chem. Int. Ed.* **2012**, *51*, 8555; *Angew. Chem.* **2012**, *124*, 8683.

S3. Grünbaum, B.; Shepherd, G. C. *Tilings and Patterns*, W. H. *Freeman* & Company, New York, **1986**.
